# Exploring the Potential Performance of Fibroscan for Predicting and Evaluating Metabolic Syndrome using a Feature Selected Strategy of Machine Learning

**DOI:** 10.3390/metabo13070822

**Published:** 2023-07-05

**Authors:** Kuan-Lin Chiu, Yu-Da Chen, Sen-Te Wang, Tzu-Hao Chang, Jenny L Wu, Chun-Ming Shih, Cheng-Sheng Yu

**Affiliations:** 1 Department of Family Medicine, Taipei Medical University Hospital, Taipei 110301, Taiwan; 2 Department of Family Medicine, School of Medicine, College of Medicine, Taipei Medical University, Taipei 11031, Taiwan; 3 Health Management Center, Taipei Medical University Hospital, Taipei 110301, Taiwan; 4 Graduate Institute of Biomedical Informatics, College of Medical Science and Technology, Taipei Medical University, Taipei 235603, Taiwan; 5 Clinical Big Data Research Center, Taipei Medical University Hospital, Taipei 110301, Taiwan; 6 Department of Internal Medicine, School of Medicine, College of Medicine, Taipei Medical University, Taipei 11031, Taiwan; 7 Cardiovascular Research Center, Taipei Medical University Hospital, Taipei 11031, Taiwan; 8 Taipei Heart Institute, Taipei Medical University, Taipei 11031, Taiwan; 9 Graduate Institute of Data Science, College of Management, Taipei Medical University, Taipei 235603, Taiwan; 10 Clinical Data Center, Office of Data Science, Taipei Medical University, Taipei 106339, Taiwan

**Keywords:** machine learning, liver steatosis, non-alcoholic fatty liver disease, controlled attenuation parameter, liver stiffness measurement, metabolic syndrome

## Abstract

Metabolic syndrome (MetS) includes several conditions that can increase an individual’s predisposition to high-risk cardiovascular events, morbidity, and mortality. Non-alcoholic fatty liver disease (NAFLD) is a predominant cause of cirrhosis, which is a global indicator of liver transplantation and is considered the hepatic manifestation of MetS. FibroScan^®^ provides an accurate and non-invasive method for assessing liver steatosis and fibrosis in patients with NAFLD, via a controlled attenuation parameter (CAP) and liver stiffness measurement (LSM or E) scores and has been widely used in current clinical practice. Several machine learning (ML) models with a recursive feature elimination (RFE) algorithm were applied to evaluate the importance of the CAP score. Analysis by ANOVA revealed that five symptoms at different CAP and E score levels were significant. All eight ML models had accuracy scores > 0.9, while treebags and random forest had the best kappa values (0.6439 and 0.6533, respectively). The CAP score was the most important variable in the seven ML models. Machine learning models with RFE demonstrated that using the CAP score to identify patients with MetS may be feasible. Thus, a combination of CAP scores and other significant biomarkers could be used for early detection in predicting MetS.

## 1. Introduction

Non-alcoholic fatty liver disease (NAFLD) is now a predominant cause of cirrhosis and an indicator for liver transplantation worldwide. It is considered the hepatic manifestation of metabolic syndrome (MetS) due to the coexistence of visceral obesity, insulin resistance, and dyslipidemia [1,2,3]. It is estimated that NAFLD, similar to obesity and diabetes, causes more than 30% of all liver diseases [4,5]. Liver biopsies are an accurate standard method for identifying patients with NAFLD and can distinguish patients with steatohepatitis from those with steatosis. However, liver biopsies are costly and have a high sampling error rate. Moreover, there is the potential for procedure-related complications, such as pain, bleeding, hemothorax, and bile peritonitis, which can increase the risk of morbidity and mortality [6,7]. Therefore, more feasible and practical detection methods are required to identify patients at a high risk of NAFLD. Non-invasive methods such as FibroScan can detect NAFLD more efficiently and provide an easier way to diagnose MetS [8,9]. Biomarkers are used to examine liver inflammation and fibrosis in daily clinical practice. However, certain proteins or biomarkers, such as γ-GT, and ALK-P, cannot be directly used to detect liver steatosis or fibrosis since they do not target a specific organ and may be associated with diseases in multiple organs [10].

MetS encompasses several conditions that could exacerbate an individual’s risk of cardiovascular events, such as heart failure and myocardial infarction or coronary artery disease, which further increases morbidity and mortality [11]. According to the National Cholesterol Education Program (NCEP) Adult Treatment Panel III (ATP III) criteria, the diagnosis of MetS requires at least three of the five following conditions: (1) abdominal obesity (also highly correlated with insulin resistance), (2) elevated triglycerides (TG), (3) reduced high-density lipoprotein (HDL) cholesterol, (4) elevated blood pressure, and (5) elevated fasting glucose (impaired fasting glucose or type 2 diabetes mellitus) [12]. Various attempts have been made to quantitatively assess MetS. One of the simpler and better-established scores for that purpose is the siMS score [13,14].

The high prevalence of MetS necessitates a large-scale screening. However, some patients may not like the discomfort and inconvenience of the fasting blood tests that are required for diagnosing MetS in clinical settings. Hence, it is reported that MetS patients remain undiagnosed [15]. FibroScan, an ultrasound-based device that utilizes patented naval technology, vibration-controlled transient elastography (VCTE^TM^), can provide an accurate and non-invasive method for assessing liver steatosis and fibrosis in patients with NAFLD, while it is currently widely used in clinical practices [16]. It uses the velocity of shear waves (also known as slow waves) to assess liver stiffness. Shear waves move transversely, perpendicular to the motion of the affected tissue. When a shear wave is applied, it quickly becomes attenuated by the liver tissue. The speed of the wave traveling through the tissue is inversely proportional to the elasticity of the tissue. This method was invented by the Langevin Institute and was initially implemented to evaluate cheese maturation. However, it has been used in medical practice since 2001 under the name *FibroScan*^®^ [17,18]. The acquired data are processed based on the physical characteristics of the shear waves and are shown as liver stiffness measurement (LSM) and controlled attenuation parameter (CAP) scores [19,20].

Artificial intelligence in ML techniques has succeeded in predicting and diagnosing numerous diseases, such as cancer and chronic kidney and liver diseases [21,22,23,24,25,26]. A decision tree algorithm has also been applied in MetS prediction model building, successfully identifying potential patients with MetS in a self-paid health examination population [27,28]. Moreover, studies have demonstrated that ML and data visualization can identify the relationships between metabolic conditions and potential risk factors for MetS and distinguish non-obese patients with MetS using CAP scores [29]. Supervised ML techniques generally improve prediction ability, whereas unsupervised learning techniques extract patterns and characteristics. Our study aimed to combine the non-invasive FibroScan technology and several ML algorithms with RFE to assess the accuracy of an AI-based diagnostic tool for MetS and identify the potential thresholds of clinical biomarkers in preventive medicine.

## 2. Materials and Methods

### 2.1. Study Design and Setting

This retrospective cohort study included healthy participants who visited the Health Management Center (HMC) at the Taipei Medical University Hospital (TMUH) for a self-paid health examination to test the ability of FibroScan and supervised ML in identifying and predicting the risk of MetS. The study was conducted at the TMUH, and the electronic medical record of each participant was reviewed. The TMUH is a private teaching hospital in Taiwan, and its HMC receives > 50 visits per day. This study was approved by the Institutional Review Board of the TMU (No: N201903080) and was conducted in accordance with the Declaration of Helsinki. Due to the retrospective nature of this study, the requirement for informed consent was waived by the Institutional Review Board. The electronic medical records were converted into an anonymous format to protect the privacy of the patients. The completeness and correctness of the participants’ self-answered questionnaires (relating to demographics and existing medical conditions) were verified by well-trained medical staff. Patient adherence to health examination prerequisites, such as overnight fasting for at least eight hours before the examination, was also verified. Those that did not meet the prerequisites were rescheduled.

### 2.2. Patient and Data Selection Criteria

Self-paid health examination participants who underwent an abdominal transient elastography inspection using the FibroScan 502 Touch (Echosens, Paris, France) and urine tests at the HMC of the TMUH between March 2015 and December 2019 were included in this study. Participants ≤ 18 years old or whose records were incomplete were excluded from the study, which resulted in the enrollment of 1944 participants. Data were collected from the participants using invasive and non-invasive methods. Information collected by non-invasive methods included (1) anthropometrics (weight, height, waist circumference, and blood pressure); (2) incidence of proteinuria, hematuria, red blood cell cast, white blood cell cast, and other urine sediment abnormalities (via urine specimens obtained in the morning and scheduled to avoid menstrual periods. If urine test results were abnormal, tests were repeated within three months); (3) The CAP score and liver stiffness parameter (E score) from the FibroScan (502 Touch; Echosens, Paris, France) test. The collection of blood samples represented the only invasive method used. Test items included albumin, globulin, cholesterol, creatinine, low-density lipoprotein (LDL) cholesterol, non–high-density lipoprotein (non-HDL) cholesterol, glycated hemoglobin (HbA1c), serum glutamic oxaloacetic transaminase (GOT), serum glutamic-pyruvic transaminase (GPT), gamma-glutamyl transferase (γGT), alkaline phosphatase (ALKp), total protein (T_protein), total bilirubin (T_bilirubin), direct bilirubin (D_bilirubin), blood urea nitrogen (BUN), uric acid (UA), estimated glomerular filtration rate (eGFR), thyrotropin (TSH), alpha-fetoprotein (AFP), and glucose Ante Cibum (glucose AC).

### 2.3. Definitions of Measurement Cutoffs and Calculations

MetS was identified based on the presence of at least 3 out of the 5 symptoms specified in the NCEP ATP III definition of MetS [19,23]: large WL (≥80 cm for women and ≥90 cm for men), high TG (≥150 mg/dL), reduced HDL levels (<50 mg/dL and <40 mg/dL for women and men, respectively), elevated blood pressure (BP; systolic BP ≥ 130 mmHg or diastolic BP ≥ 85 mmHg), and increased fasting blood sugar (≥100 mg/dL) or use of medication to control the latter 4 of these conditions. Cutoff points were adopted from the NCEP ATP III definition with ethnic-specific cutoff points for waist circumference and an equality principle on the five disorders [22]. The FibroScan CAP and E score levels are described in Table S1.

### 2.4. Statistical Analysis and Machine Learning

Statistical analyses were performed using R Statistical Software (v 4.2.2, R Core Team 2021). Analysis of variance (ANOVA) tests were used to compare the means of the different groups. The objective of the experiment was to investigate various factors that might affect the outcomes of the participants’ health conditions, thereby controlling both the average level and quality variability. In all analyses, *p* < 0.05 was considered statistically significant [30].

In this study, we applied several ML models to evaluate their performance in diagnosing MetS. We summarize the ML models used in this study in Supplementary Materials Table S3. The information and settings of the hyperparameters can be found in reference [31], and the details can be found on the caret Package’s website [32]. According to the ‘caret’ package in R, cross-validation eliminates features from a model. Recursive feature elimination (RFE) is achieved by fitting the model multiple times and, at each step, removing the weakest features determined by either the coefficients or important feature attributes in the model. To find the optimal number of features, the number of features with the highest cross-validated test score was selected for each machine learning model.

Many statistical or ML models are available in this package. ‘rfFuncs’ (random forests) uses a random forests method of assessing the mean decrease in accuracy over the features of interest. ‘ldaFuncs’ (LDA) applies a linear discriminant analysis for classification only (also called Fisher’s linear discriminant), which is used to find a linear combination of features that separates two or more classes of objects or participants. ‘nbFuncs’ (Naïve Bayes) uses the Naïve Bayes algorithm to assess the features that have the greatest effect on the overall probability of the dependent variable. ‘treebagFuncs’ (treebags) explains how many times a variable occurs as a decision node. The number of occurrences and position of a given decision node in the tree indicate the importance of the respective predictor, and the closer a decision node is to the root node, the more important the variable. ‘lrFuncs’ (LR) uses logistic regression for classification, which is the typical statistical learning model for prediction. The other three methods, support vector machines (SVM), neural network (nnet), and classification and regression tree (CART), are available in the function ‘train.’ They can be applied to ‘caret’ with their specific tuning parameters (more details can be obtained from ‘getModelInfo’ in R). SVM uses a linear kernel to analyze the data for classification and regression analysis. It maps the participants in the training set to points in space to maximize the gap width between the two categories of patients. nnet fits a single-hidden-layer neural network to avoid the disadvantage of black-box issues and to construct an appropriate classification neural network with one output and an entropy fit if the number of levels is two for the outcome of MetS. CART is the typical decision tree model from the ‘rpart’ library, which uses the Gini index as the metric and measures the distribution among field-specific afflictions, to predict the patients with MetS [31].

The number of selected features and the performance of each ML algorithm are assessed by many criteria from the confusion matrix provided by the ‘caret’ package. In the performance evaluation, accuracy essentially indicates where, among all references, the proportion is mapped correctly. The overall accuracy is usually expressed as a percentage, with 100% being a perfect classification, where all reference sites were classified correctly. The kappa coefficient evaluates how well the classification performed compared to randomly generated assigned values. In this test, the kappa indicates the agreement between frequencies of the categorical data and what would be expected by chance. A value of 0 indicates that the classification is not better than a random classification, although a value close to 1 indicates that the classification is significantly better than the random classification. Kappa is an excellent performance measure when the classes are highly unbalanced. This study applied recursive feature selection to each ML model with 10-fold cross-validation, which was repeated 5 times as the outer resampling method [33,34,35,36]. The formulae for the criteria in the confusion matrix are described in Table S2.

## 3. Results

**Figure 1:**
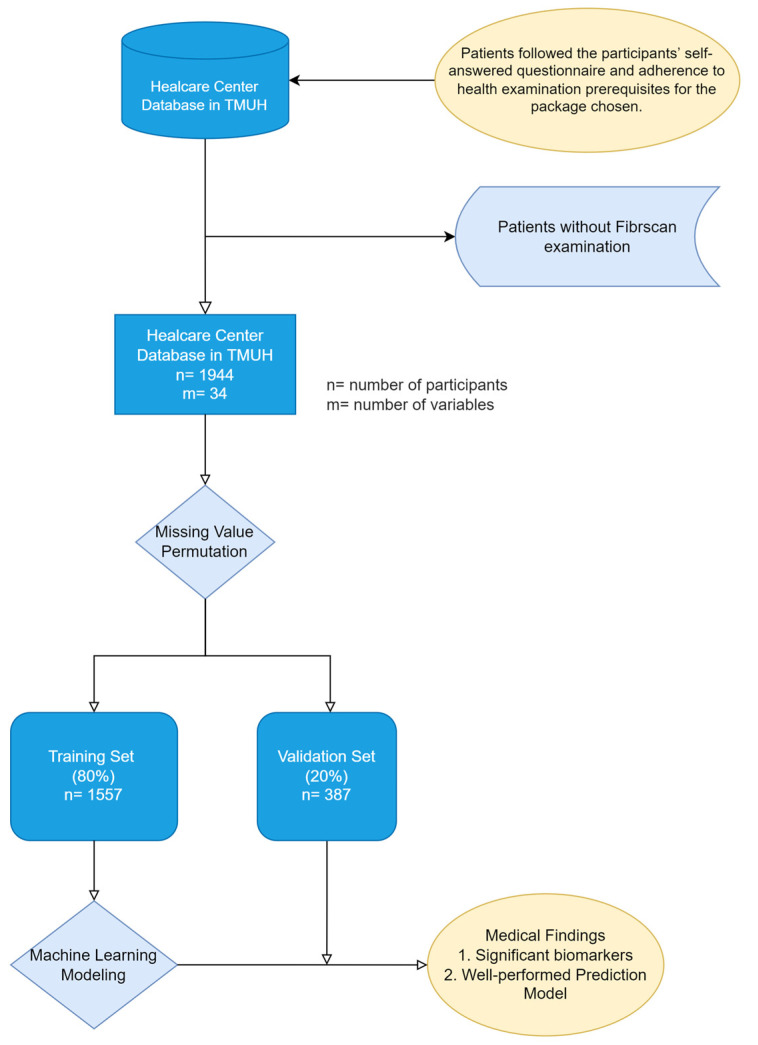
Flowchart of data collection and preprocessing for machine learning analysis in this study. Here, ‘n’ means the number of participants and ‘m’ means the number of variables used as the risk factors.

**Figure 2:**
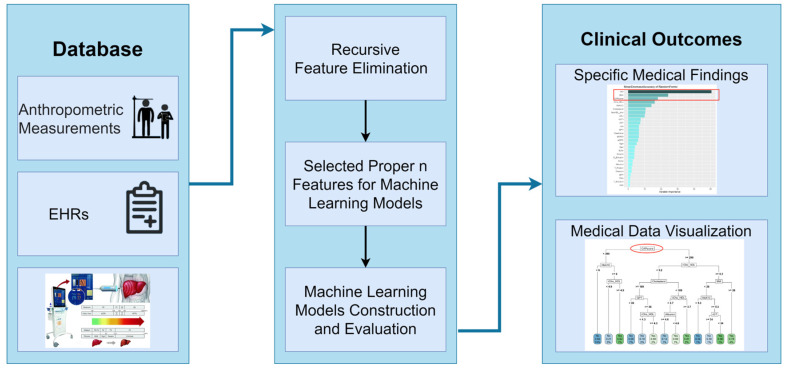
The structure of AI-based strategy with multi-model data and machine learning techniques for metabolic syndrome detection.

Figure 1 illustrates the data collection procedure at the TMUH HC, with the subsequent data preprocessing for the ML analysis. Data inclusion and exclusion, missing value permutations, training and testing set division with independence, and model construction are presented. The systematized analysis, which includes a multi-model database, ML modeling with RFE, and clinical outcomes from the ML pipeline, is depicted in Figure 2.

Table 1 shows the ANOVA results for the five MetS symptoms at different levels of the CAP and E scores measured by FibroScan. The CAP and E scores were classified into four levels based on the definition by the FibroScan manual. Each symptom of MetS was significant for both the CAP and E score levels. At least three p-values for the five symptoms were considered extremely significant. Apart from albumin, T_bilirubin, and D_bilirubin, all clinical factors were significant (only four were not extremely significant; Table 2).

**Figure 3:**
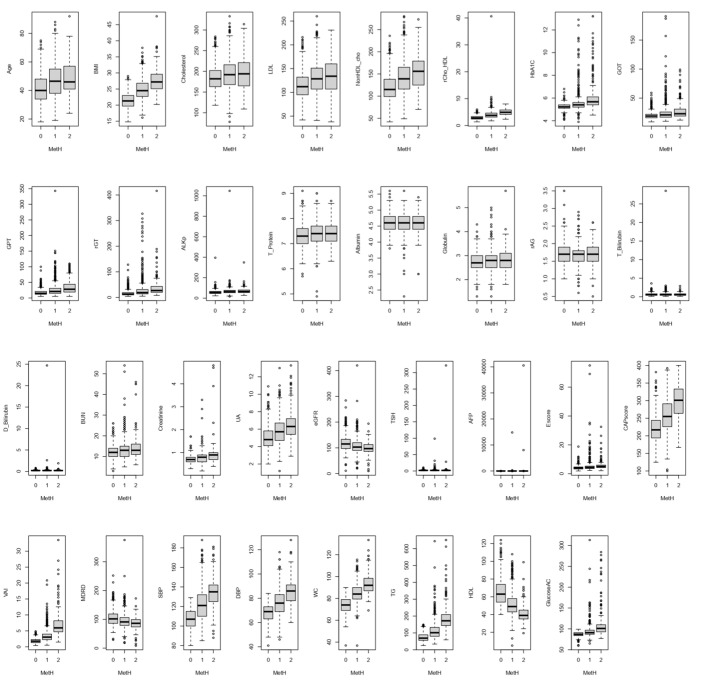
A boxplot for each clinical biomarker compared between different metabolic health conditions. The small circle (○) is noted as outliers in each boxplot.

**Figure 4:**
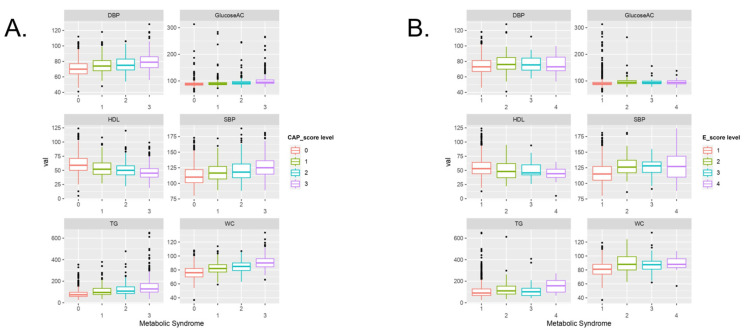
Boxplots of five metabolic symptoms to compare the different levels of (**A**) CAP scores and (**B**) E scores.

The box depicts the difference between the symptoms in patients with MetS for all the clinical biomarkers in Figure 3 (a box plot, excluding the outliers, is shown in Supplementary Materials Figure S1). Moreover, variations in the five symptoms of MetS at each level of CAP and E scores are also depicted by the box plot in Figure 4.

MetH denotes the metabolic health condition with three levels. ‘0’ indicates none of the five symptoms in this level, ‘1’ means patients reach one or two of the five symptoms in this level, and ‘2’ means patients reach at least three of the five symptoms in this level.

**Figure 5:**
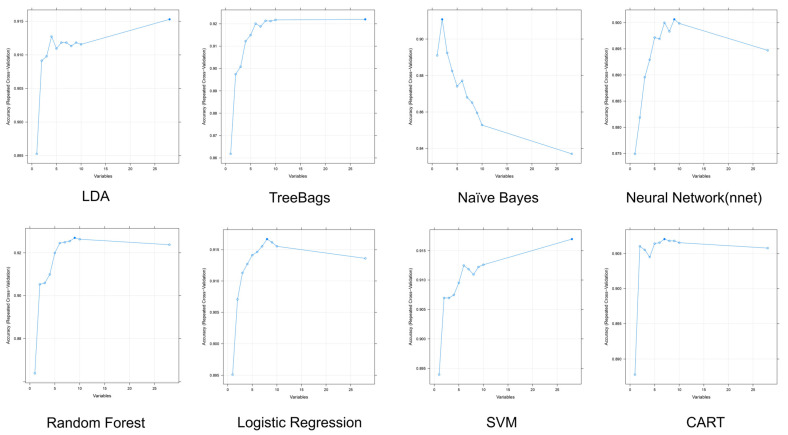
The variations in accuracy with different numbers of variables are involved in each machine learning model. The *y*-axis plots the cross-validated test score, while the *x*-axis plots the number of features.

After a series of data preprocessing steps, the training dataset was applied to ML modeling with RFE. Table 3 and Figure 5 illustrate the overall outcomes of the RFE algorithm for several ML models. The optimal number of variables, best performance, and rankings of the top factors are listed in the table. The accuracy of all 8 models was >0.9. Moreover, the treebags and random forest models achieved kappa values of 0.6439 and 0.6533, respectively. However, the random forest method had the best accuracy and kappa scores, whereas SVM was the least variable for both scores. LDA, treebags, and SVM achieved the best performance when all risk factors were involved; however, Naïve Bayes only used two variables as predictors. The variation in accuracy with different numbers of variables for each model is depicted in detail in Figure 5.

**Figure 6:**
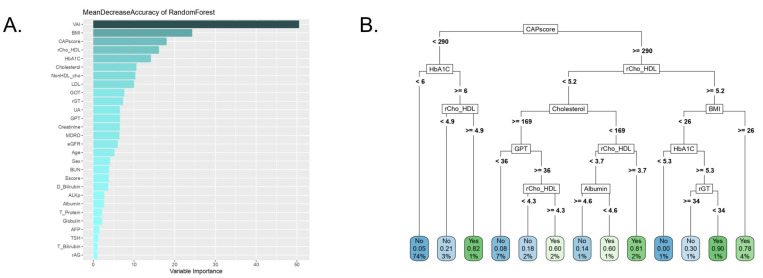
A visualization with (**A**) gradient coloring for variable importance rank and (**B**) decision tree.

Figure 6 depicts the decision tree and how the variables rank in importance by random forest. The CAP score features among the three most important factors (Figure 6A). After excluding the top two variables, the decision tree model indicated a threshold of 290 for the CAP score at the root node in the CART model (Figure 6B).

Finally, the overall performances of the eight ML models with all risk factors are listed in Table 4. The random forest and SVM models were the most accurate (above 0.90). However, nnet was the most sensitive (0.6170), while SVM and CART had the best specificity scores (> 0.96). Moreover, treebags and random forest achieved the highest kappa scores (0.5322 and 0.5480, respectively), whereas SVM was the most precise (0.6571). A comparative analysis between traditional statistical methods and AI machine learning methods can be found in Supplementary Materials Figure S2. Using traditional logistic regression on the CAP and E score combination (FibroScan (Echosens, Paris, France)), the receiver operating characteristic curve (ROC) was 0.7743. However, the ROC can be greatly enhanced to ~0.91–0.93 by AI machine learning methods.

## 4. Discussion

FibroScan is a non-invasive device originally designed to measure liver stiffness (E score) and fatty liver (CAP score) [16]. Fatty liver was reported as a component of MetS in 2008, and the CAP score was also subsequently found to be associated with MetS [37,38]. In this study, we found that every symptom of MetS was significant within the different levels of either the CAP or E scores. In addition, a combination of the CAP and E scores can be used to detect MetS with moderate accuracy (ROC of 0.7743). Using ML models, we can further improve the accuracy of MetS detection to 0.93. Hence, it is valuable to establish an appropriate model with feature selection and ML for MetS, as many of the clinical biomarkers are very similar to the five symptoms of MetS.

To identify potential biomarkers for prediction, the best combinations of clinical biomarkers were identified after a ML analysis using RFE. Based on cross-validation with different numbers of risk biomarkers, the treebags and random forest models reached a plateau in accuracy when the number of variables exceeded seven, which explored their power for prediction when finite crucial risk factors were involved. Although Naïve Bayes achieved the best performance with the fewest variables, its performance declined as the number of variables increased. This was related to collinearity and interference from dependent variables. While logistic regression and nnet also demonstrated a small reduction in accuracy as the number of variables increased, the loss in accuracy was almost negligible, thereby resulting in minimal bias. LDA and SVM with a linear kernel exhibited the same linear trend in accuracy when the number of variables increased. This indicates that the linear combination can explain more information in space when the number of dimensions increases.

The visceral adiposity index (VAI) is a scoring system based on WC, TG, and HDL [39]. It indicates visceral adiposity dysfunction and insulin sensitivity [40] and has become a useful tool for assessing MetS and identifying high-risk patients [41]. The BMI is the most used parameter to monitor obesity and can be calculated using self-reported height and weight. Obesity is associated with various diseases, including diabetes, cancer, and hypertension [42]. Moreover, it is associated with the risk of NAFLD and MetS due to the overlap in the occurrence of other risk factors, such as atherosclerosis, type 2 diabetes, and hypertension [43]. Therefore, the VAI and BMI remain influential because obesity is highly related to MetS. Detecting MetS in patients with a non-obese phenotype is challenging in clinical practice. However, a previous study has revealed that the CAP score can potentially address this issue [29,44]. Since the CAP score was the top variable in seven of the eight ML models in this study, it may be capable of detecting MetS because it reflected the severity of fatty liver disease in those patients.

The CAP score reflects fat accumulation in the liver and the degree of steatosis [45]. A fatty liver is an important component of MetS in that it is highly prevalent in patients with MetS. Furthermore, all MetS conditions correlate with liver fat [38]. Moreover, liver fat content influences fasting serum insulin, C-peptide, and other MetS-related factors [46]. Therefore, a fatty liver condition should be considered when discussing MetS. This also highlights that the CAP score could be a strong predictor of MetS, as it ranked as one of the top variables from the feature selection of several ML models.

Patients with MetS and abdominal obesity usually develop atherogenic dyslipidemia [47]. Therefore, dyslipidemia-related features, such as HDL, non-HDL, and cholesterol were found to be significant in detecting MetS. Independently, HDL levels are reported to be associated with insulin sensitivity [48] and, combined with TG measurements, can be used to measure insulin-mediated glucose disposal [49]. The cholesterol/HDL ratio (Chol/HDL) has also been associated with MetS [50]. Non-HDL cholesterol was also found to be significant in assessing MetS: non-HDL was reported to be higher in patients with MetS, and it was suggested that non-HDL was a better predictor than LDL [51]. However, since a low level of HDL was used as one of the criteria for defining MetS, it is obvious that HDL will be the best predictor of MetS for dyslipidemia-related features.

Chronic hyperglycemia triggers and indicates that dysmetabolism may lead to MetS [52]. Therefore, the serum glucose level is a metabolic parameter that can be used to assess MetS [53]. The American Diabetes Association (ADA) recommends the use of HbA1c as an indicator of increased diabetes risk because it is not limited to fasting samples and can reflect average glycemia over a long period, which is better than glucose AC [54]. In addition, some studies have shown that elevated HbA1c levels are associated with dysmetabolism [55,56].

γGT levels reflect the degree of liver damage and alcohol consumption and have been found to correlate with MetS-related illnesses, such as diabetes, hypertension, and cardiovascular mortality, regardless of their relationship with liver damage [57,58]. Studies have also shown that elevated γGT concentrations correlate to the prevalence of MetS [59,60]. This phenomenon may occur because γGT indirectly reflects the elevation in inflammation and oxidative stress induced by dysmetabolism [61].

## 5. Limitations

This study has several limitations. Firstly, this was a retrospective study, which may have included selection bias. Hence, this study should be further validated by a prospective study. Secondly, this study enrolled 1944 Taiwanese patients from the same hospital. It is necessary to further validate this study using an external dataset with a bigger sample size, and with different racial demographics. Currently, the effectiveness and robustness of our strategy, when applied to a different hospital or different racial demographics is unclear. Thirdly, this study does not include alcohol consumption, as well as chronic hepatitis B/C variables. Alcohol consumption and chronic hepatitis B/C might well lead to liver fibrosis and cirrhosis, both being characterized by increased liver stiffness. Thus, the omission of the impact of the widely detected alcohol consumption and chronic hepatitis B/C in the FibroScan-assisted assessment of MetS might severely influence the validity of the proposed model. However, we think there is a limited number of liver fibrosis patients. A previous study suggested that the FibroScan E score can be used to detect liver fibrosis patients, and we found that there was only 6% of patients (124/1944) with an E score that was indicative of fibrosis [62,63].

## 6. Conclusions

We demonstrated, via ML models with RFE and data visualization, that the CAP score could be used to identify patients with MetS. We also showed that a combination of the CAP score and some potential risk factors could represent various health conditions associated with MetS and provide a precise prediction model for the complicated relationship between metabolic symptoms and their comorbidities for early detection in the future.

## Figures and Tables

**Table 1:** Descriptive statistics and ANOVA for each different stage of CAP score and E score in Fibroscan examinations.

	CAP Score				
	S0 (739)	S1 (245)	S2 (252)	S3 (320)	*p*-Value
DBP, mmHg	71.2	74.6	75.9	79.8	<0.001 *
	70 (64–77)	74 (68–81)	75 (69–83)	79 (72–86)	
SBP, mmHg	112.5	121.1	121.1	126	<0.001 *
	110 (101–122)	118 (109–131)	118 (109–131)	125 (115–136)	
HDL, mg/dL	61	54.3	51.2	46.7	<0.001 *
	59 (50–71)	52 (43–63)	50 (42–58)	45 (38–53)	
Glucose AC, mg/dL	89.2	92.6	95.5	101.4	<0.001 *
	88 (84–92)	89 (86–94)	92 (88–98)	95 (91–104)	
TG, mg/dL	83.4	108.7	119.3	149.7	<0.001 *
	74 (58–97)	95 (77–133)	108 (86–148)	128 (99–178)	
WC, cm	76.2	82.3	85.2	91	<0.001*
	76 (70–82)	82 (77–87.5)	85 (79–90)	90 (84.5–96)	
	E score				
	F0–F1 (1462)	F2 (50)	F3 (19)	F4 (25)	*p*-value
DBP, mmHg	74.1	77.8	77.2	75.8	0.0162
	73 (67–81)	76 (70–85)	76 (69–84)	73 (68–86)	
SBP, mmHg	117	127.9	126.7	127.9	<0.001 *
	115 (105–127)	126 (117–137)	128 (118–134)	127 (110–144)	
HDL, mg/dL	55.7	50	50.9	44.9	<0.001 *
	53 (44–64)	48 (37–62)	46 (42–60)	44 (38–52)	
Glucose AC, mg/dL	93	99.2	98	96.9	0.0069
	90 (86–95)	95 (90–101)	94 (90–99)	94 (89–101)	
TG, mg/dL	105.5	128.1	125.8	161.1	<0.001 *
	90 (67–128)	110 (81–154)	101 (67–134)	157 (99–206)	
WC, cm	81.2	89.9	89	89.6	<0.001 *
	81 (74–88)	88 (80–99)	87.5 (81–92.5)	88 (83.5–96)	

The statistics in the table denote the mean and median (Q1–Q3) in the first and second columns, respectively. The number of patients in each stage is shown in brackets. The abbreviations and full names of every factor list are as follows: CAP score, controlled attenuation parameter score; DBP, diastolic blood pressure; SBP, systolic blood pressure; cholesterol; HDL, high-density lipoprotein cholesterol; glucose AC, glucose Ante Cibum; TG, triglycerides; WC, waist circumference. * depicts the *p*-value as extremely significant.

**Table 2:** Descriptive statistics and ANOVA for different groups of metabolic syndrome health conditions.

	Metabolic Syndrome Health Conditions			
	C (5, 0); N_1_ = 753	C (5, 1 and 2); N_2_ = 938	C (5, 3, 4, and 5); N_3_ = 253	*p*-Value
Age, years	40.7; 40 (34–48)	47; 47 (38–55)	48.7; 46 (41–57)	<0.001 *
BMI, kg/m^2^	21.4; 21.3 (19.7–23)	24.9; 24.5 (22.7–26.8)	27.6; 27.2 (25.1–29.6)	<0.001 *
Cholesterol, mg/dL	184.2; 182 (163–202)	193.1; 192 (168–216)	194.3; 194 (165–221)	<0.001 *
LDL, mg/dL	114.9; 112 (95–132)	129.9; 129 (107–151)	132.4; 134 (107–160)	<0.001 *
non-HDL, mg/dL	119.2; 115 (99–138)	141.6; 139 (116–165)	153.9; 156 (125–179)	<0.001 *
Chol/HDL	2.95; 2.83 (2.42–3.35)	3.99; 3.79 (3.15–4.64)	4.98; 4.93 (4.11–5.81)	<0.001 *
HbA1c, %	5.2; 5.2 (5.1–5.4)	5.5; 5.4 (5.2–5.6)	6.1; 5.7 (5.4–6.1)	<0.001 *
GOT, U/L	19.9; 19 (16–22)	23.8; 21 (17–26)	27.1; 23 (19–31)	<0.001 *
GPT, U/L	17.9; 15 (12–21)	26.5; 21 (15–31)	35.3; 28 (19–44)	<0.001 *
γGT, U/L	16.2; 13 (10–19)	28.3; 19 (14–31)	36.8; 27 (20–43)	<0.001 *
ALKp, IU/L	58; 55 (46–66)	67.6; 64 (53–76)	69.8; 65 (55–80)	<0.001*
T_Protein, g/dL	7.4; 7.3 (7–7.6)	7.4; 7.4 (7.1–7.7)	7.42; 7.4 (7.1–7.7)	0.0259
Albumin, g/dL	4.6; 4.6 (4.4–4.8)	4.6; 4.6 (4.4–4.8)	4.6; 4.6 (4.4–4.8)	0.157
Globulin, g/dL	2.73; 2.7 (2.5–3.0)	2.79; 2.8 (2.5–3.0)	2.82; 2.8 (2.5–3.1)	0.0006
Alb/Glb	1.73; 1.7 (1.5–1.9)	1.69; 1.7 (1.5–1.8)	1.68; 1.7 (1.5–1.9)	0.00265
T_Bilirubin, mg/dL	0.64; 0.6 (0.4–0.8)	0.68; 0.6 (0.4–0.8)	0.67; 0.6 (0.4–0.8)	0.319
D_Bilirubin, mg/dL	0.23; 0.2 (0.2–0.3)	0.26; 0.2 (0.2–0.3)	0.24; 0.2 (0.2–0.3)	0.561
BUN, mg/dL	12.2; 12 (10–14)	13.4; 13 (10–15)	14; 13 (11–16)	<0.001 *
Creatinine, mg/dL	0.70; 0.7 (0.6–0.8)	0.78; 0.8 (0.6–0.9)	0.89; 0.9 (0.7–1.0)	<0.001 *
UA, mg/dL	5.02; 4.8 (4.1–5.8)	5.78; 5.7 (4.7–6.7)	6.4; 6.3 (5.4–7.2)	<0.001 *
eGFR, ml/min/1.73 m^2^	117.4; 115 (97–133)	107.1; 102.9 (90–120)	99.1; 97 (86–113)	<0.001 *
TSH, mU/L	2.00; 1.80 (1.23–2.49)	2.25; 1.77 (1.21–2.48)	3.36; 1.75 (1.28–2.42)	0.0328
AFP, ng/mL	2.60; 2.26 (1.59–3.11)	18.98; 2.41 (1.69–3.36)	194.93;2.43 (1.74–3.19)	0.0272
E score, kPa	4.2; 4.0 (3.3–4.7)	5.0; 4.4 (3.6–5.3)	5.6; 5.0 (4.3–6.0)	<0.001 *
CAP score, dB/m	220.1; 217 (194–244)	259; 255 (226–291)	298.3; 301 (264–333)	<0.001 *
VAI	1.88; 1.77 (1.32–2.30)	3.41;3.05 (2.27–4.00)	6.94; 5.95 (4.73–8.19)	<0.001 *
MDRD	104; 102 (86–119)	95;91 (79–106)	87; 86 (74–99)	<0.001 *
SBP, mmHg	107.2; 107 (100–115)	121.6; 121 (110–132)	134; 135 (125–142)	<0.001 *
DBP, mmHg	68.3; 69 (63–73)	76.3; 76 (69–83)	84.9; 86 (78–91)	<0.001 *
WC, cm	74.2; 74 (69–79)	84.7; 84 (79–90)	93.2; 92 (87–98.5)	<0.001 *
TG, mg/dL	73.7; 69 (56–89)	112.8; 101 (80–133)	187.4; 172 (144–209)	<0.001 *
HDL, mg/dL	65.1; 63 (54–74)	51.5; 49 (43–58)	40.4; 39 (35–45)	<0.001 *
Glucose AC, mg/dL	87.3; 87 (84–91)	93.9; 91 (87–97)	109.5; 101 (93–110)	<0.001 *

The statistics in the table denote the mean; median (Q1–Q3) for each group. The denotation of metabolic syndrome health condition is the combination of five symptoms. For example, C (5, 0) means patients reach none of the five symptoms, and C (5, 1 and 2) means the patients reach one or two of the five symptoms. The abbreviations and full names of every factor are as follows: BMI, body mass index; LDL, low-density lipoprotein cholesterol; non-HDL, non–high-density lipoprotein cholesterol; Chol/HDL, cholesterol/high-density lipoprotein cholesterol ratio; HbA1c, glycated hemoglobin; GOT, serum glutamic oxaloacetic transaminase; GPT, serum glutamic-pyruvic transaminase; γGT, gamma-glutamyl transferase, ALKp, alkaline phosphatase; T_Protein, total protein; Alb/Glb, albumin/globulin ratio; T_Bilirubin, total bilirubin; D_Bilirubin, direct bilirubin; BUN, blood urea nitrogen; UA, uric acid; eGFR, creatinine, estimated glomerular filtration rate; TSH, thyrotropin; AFP, alpha-fetoprotein; CAP score, controlled attenuation parameter score; VAI, visceral adiposity index; MDRD, modification of diet in renal disease formula; SBP, systolic blood pressure; DBP, diastolic blood pressure; WC, waist circumference; TG, triglycerides; HDL, high-density lipoprotein cholesterol; glucose AC, glucose Ante Cibum. * indicates the p-value as extremely significant.

**Table 3:** The number of optimal models involving clinical variables and their performances for predicting metabolic syndrome with recursive feature elimination algorithms in several machine learning models.

Model	# of Variables	Accuracy	Kappa	Accuracy SD	Kappa SD	Lists of Variables by Order *
LDA	28	0.9153	0.5772	0.01792	0.1005	VAI, BMI, Chol/HDL, CAP score, γGT, HbA1C, GPT, E score, UA, non-HDL, etc.
					
TreeBags	28	0.9220	0.6439	0.01827	0.0807	VAI, BMI, CAP score, HbA1C, Chol/HDL, cholesterol, non-HDL, LDL, Age, γGT, etc.
					
Random forest	9	0.9270	0.6533	0.01722	0.08523	VAI, BMI, CAP score, Chol/HDL, HbA1C, cholesterol, γGT, non-HDL, LDL
					
Logistic	8	0.9167	0.5928	0.01645	0.08558	VAI, BMI, Age, HbA1C, cholesterol, CAP score, non-HDL, GOT
					
Naïve Bayes	2	0.9108	0.4622	0.01693	0.09379	VAI, BMI
					
nnet	9	0.9006	0.5141	0.02340	0.1743	CAP score, VAI, AFP, cholesterol, Chol/HDL, non-HDL, γGT, eGFR, TSH
					
SVM	28	0.9170	0.5902	0.01638	0.08548	VAI, BMI, Chol/HDL, CAP score, γGT, HbA1C, GPT, E score, UA, non-HDL, etc.
					
CART	7	0.9071	0.5216	0.01864	0.1141	VAI, BMI, CAP score, Chol/HDL, HbA1C, γGT, E score
					

The abbreviations and full names of every factor are as follows: BMI, body mass index; LDL, low-density lipoprotein cholesterol; non-HDL, non–high-density lipoprotein cholesterol; Chol/HDL, cholesterol/high-density lipoprotein cholesterol ratio; HbA1c, glycated hemoglobin; GOT, serum glutamic oxaloacetic transaminase; GPT, serum glutamic-pyruvic transaminase; γGT, gamma-glutamyl transferase; UA, uric acid; eGFR, creatinine, estimated glomerular filtration rate; TSH, thyrotropin; AFP, alpha-fetoprotein; CAP score, controlled attenuation parameter score; VAI, visceral adiposity index; HDL, high-density lipoprotein cholesterol. * The order is followed by the ranking of variable importance for prediction. The first one is the top one as a predictor.

**Table 4:** The performance of different machine learning models on predicting metabolic syndrome using whole risk factors for balanced comparisons in the confusion matrix.

Model	Accuracy	Kappa	Sensitivity	Specificity	F1-Score	Precision
LDA	0.8892	0.4747	0.5319	0.9384	0.5376	0.5435
TreeBags	0.8995	0.5322	0.5957	0.9414	0.5895	0.5833
Random forest	0.9046	0.5480	0.5957	0.9472	0.6022	0.6087
Logistic	0.8969	0.5068	0.5532	0.9443	0.5652	0.5778
Naïve Bayes	0.8686	0.4297	0.5532	0.9120	0.5049	0.4643
nnet	0.8918	0.5181	0.6170	0.9296	0.5800	0.5472
SVM	0.9072	0.5103	0.4894	0.9648	0.5610	0.6571
CART	0.8995	0.4640	0.4468	0.9619	0.5185	0.6177

The dataset is initially divided into 80% and 20%, which represent the training set and testing set, respectively, while the two sets are independent. The machine learning models are established by the training set, while the performance is assessed by the testing set. In the confusion matrix of performance, all the formula criteria are described in the supplement data, including the F1-score, which constitutes the harmonic mean of precision and recall. Therefore, the performance is used to reach the balance in comparison between the eight machine learning models, while using the same independent testing set with the same number of risk factors as predicting variables.

## Data Availability

The datasets generated for this article are not publicly available due to confidentiality concerns/ethical restrictions.

## Supplementary Materials

The following supporting information can be downloaded at: https://www.mdpi.com/article/10.3390/metabo13070822/s1, Figure S1: The box plot depicts the difference between different conditions of MetS patients with whole clinical biomarkers without outliers.; Figure S2: The receiver operating characteristic curve of various AI machine learnings methods compared with other traditional scoring models; Table S1: Stages of two scores from Fibroscan; Table S2: Confusion matrix for machine learning performance criteria; Table S3: Summary of parameters of machine learning model available in caret package in R.
