# Supplementary material for: Exploring the Potential Performance of Fibroscan for Predicting and Evaluating Metabolic Syndrome using a Feature Selected Strategy of Machine Learning
Source: Metabolites. 2023 Jul 5;13(7):822. doi: 10.3390/metabo13070822 (PMC10383149; doi:10.3390/metabo13070822)
Supplement: Supplementary file 1 [file metabolites-13-00822-s001.zip › Supplementary_revised.pdf]

Table S1. Stages of two scores from Fibroscan

Parameter/Stage	Stage 1	Stage 2	Stage 3	Stage 4
CAP score	S0	S1	S2	S3
	100-238	238-260	260-290	>290
E score	F0-F1	F2	F3	F4
	2-7	7-9.5	9.5-12.5	>12.5

Table S2. Confusion matrix for machine learning performance criteria

2x2 table	True Reference Yes	True Reference No
Predicted Yes	A (TP)	B (FP)
Predicted No	C (FN)	D (TN)

TP: True positive; TN: true negative; FP: false positive; FN: false negative

The formulas of each criterion used in caret package in R programming are as follows:

$$\text{Sensitivity} = A/(A + C), \quad (1)$$

$$\text{Specificity} = D/(B + D) \quad (2)$$

$$\text{Prevalence} = (A+C)/(A+B+C+D) \quad (3)$$

$$\text{PPV} = (\text{sensitivity} * \text{prevalence})/((\text{sensitivity} * \text{prevalence}) + ((1 - \text{specificity}) *(1- \text{prevalence}))), \quad (4)$$

$$\text{NPV} = (\text{specificity} * (1 - \text{prevalence}))/(((1 - \text{sensitivity}) * \text{prevalence}) + ((\text{specificity}) *(1 - \text{prevalence}))), \quad (5)$$

$$\text{Detection Rate} = A/(A + B+C + D), \quad (6)$$

$$\text{Detection Prealence} = (A + B)/(A+B+C+ D), \quad (7)$$

$$\text{Balanced Accuracy} = (\text{sensitivity} + \text{specificity})/2, \quad (8)$$

$$\text{Precision} = A/(A + B), \quad (9)$$

$$\text{Recall} = A/(A + C), \quad (10)$$

$$F1 = (1 + beta2)* \text{precision} * \text{recall}/((beta2* \text{precision}) + \text{recall}) \quad (11)$$

,where beta = 1 for this function.

Table S3. Summary of parameters of machine learning model available in caret package in R

Model	Method Value	Type	Libraries	Tuning Parameters
LDA	lda	Classification	MASS	None
Bagged decision trees	treebag	Classification, Regression	ipred, plyr, e1071	None
Random Forest	rf	Classification, Regression	randomForest	mtry
Logistic Regression	glm	Classification, Regression	base	family = "binomial"
NaïveBayes	naive_bayes	Classification	naivebayes	laplace, usekernel, adjust
Neural Network	nnet	Classification, Regression	nnet	size, decay
SVM	svmLinear	Classification, Regression	kernlab	C
CART	rpart	Classification, Regression	rpart	cp

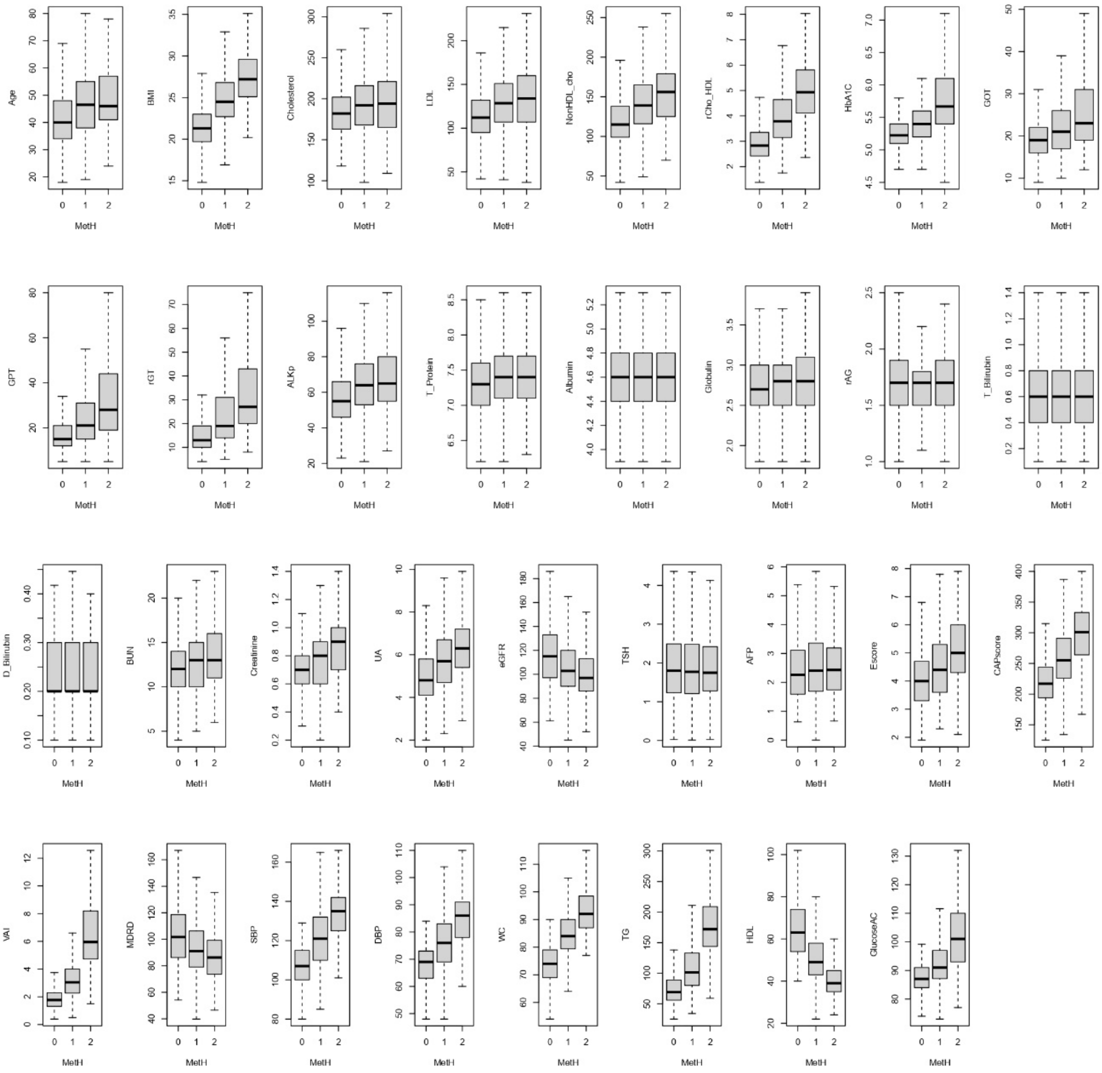

Figure S1. The box plot depicts the difference between different conditions of MetS patients with whole clinical biomarkers without outliers.

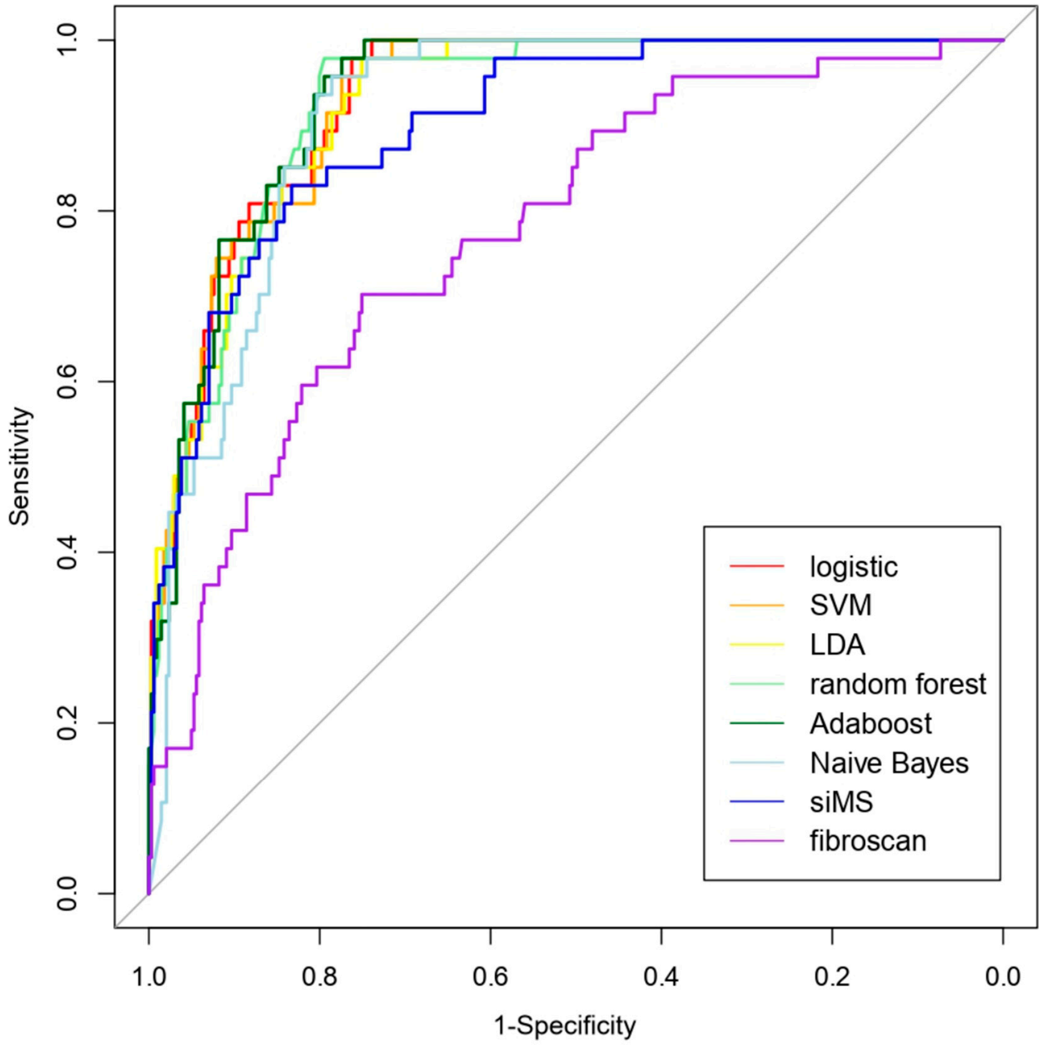

Figure S2. The receiver operating characteristic curve of various AI machine learnings methods compared with other traditional scoring models.
