# Supplementary figures and images for: Exploring the Potential Performance of Fibroscan for Predicting and Evaluating Metabolic Syndrome using a Feature Selected Strategy of Machine Learning
Source: Metabolites. 2023 Jul 5;13(7):822. doi: 10.3390/metabo13070822 (PMC10383149; doi:10.3390/metabo13070822)

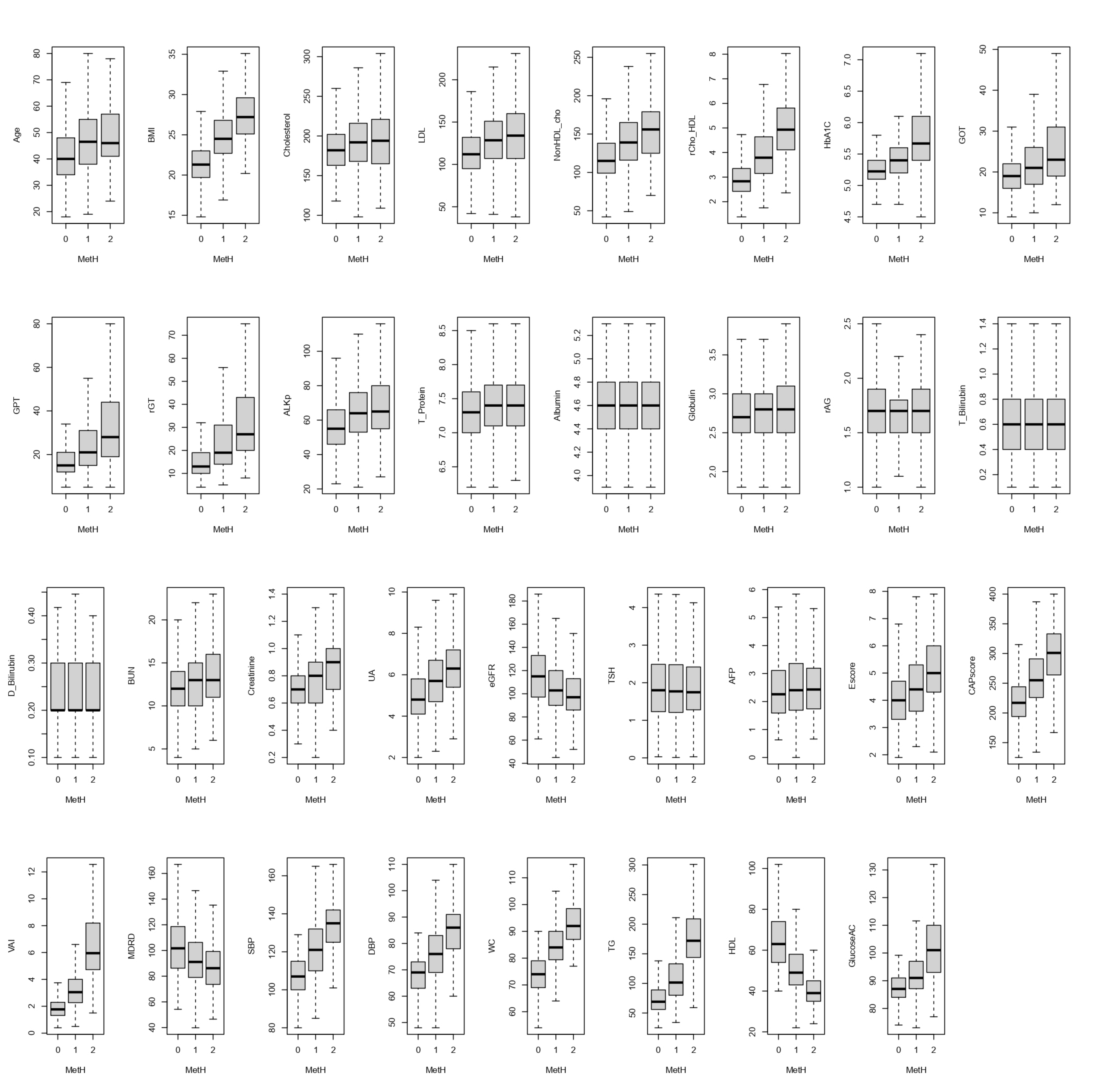

Supplement: Supplementary file 1 [file metabolites-13-00822-s001.zip › Figure_S1.png]

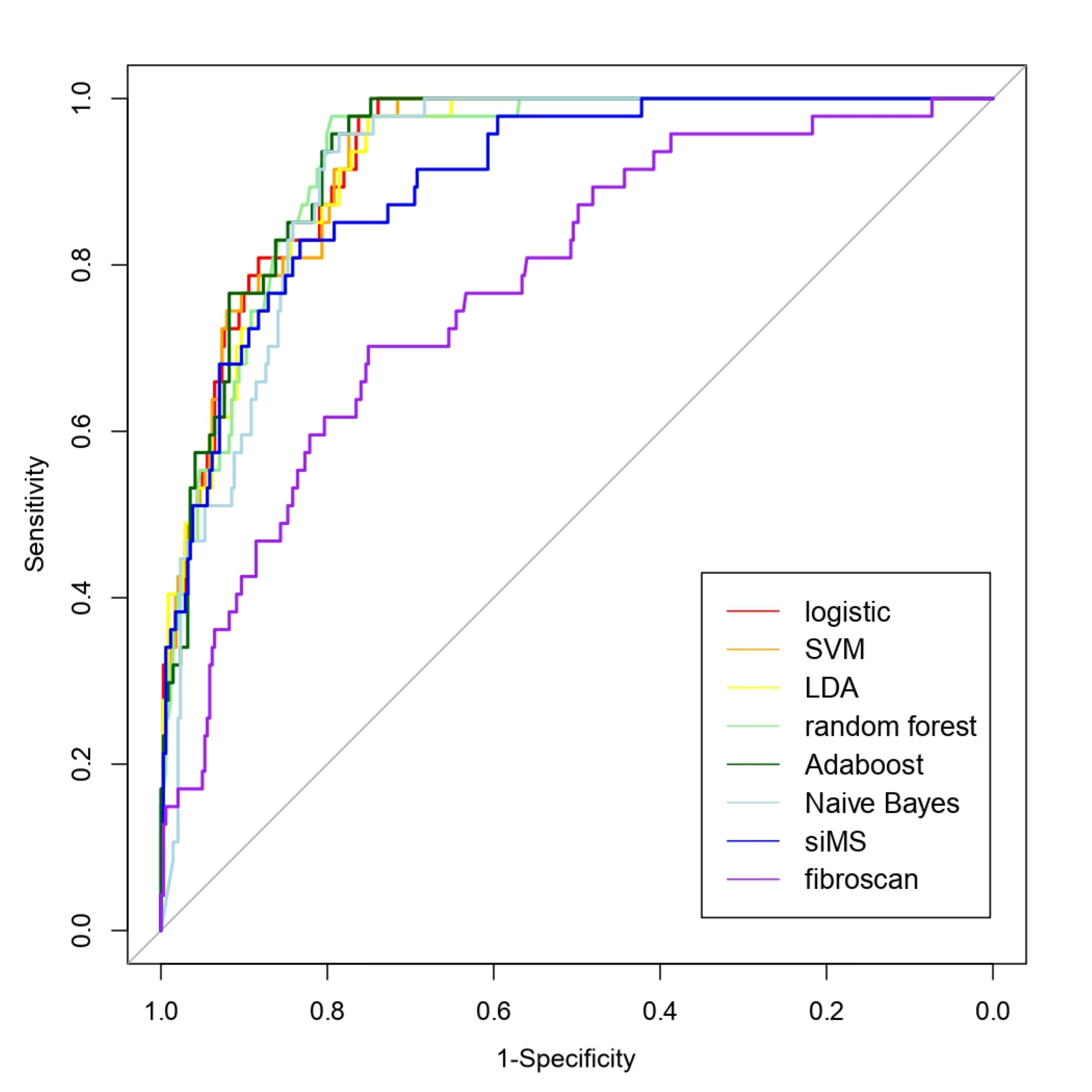

Supplement: Supplementary file 1 [file metabolites-13-00822-s001.zip › Figure_S2.png]
